# Simultaneous and Sensitive Detection of Three Pesticides Using a Functional Poly(Sulfobetaine Methacrylate)-Coated Paper-Based Colorimetric Sensor

**DOI:** 10.3390/bios13030309

**Published:** 2023-02-22

**Authors:** Jingyang Zhu, Xinru Yin, Weiyi Zhang, Meilian Chen, Dongsheng Feng, Yong Zhao, Yongheng Zhu

**Affiliations:** 1 Laboratory of Quality & Safety Risk Assessment for Aquatic Products on Storage and Preservation (Shanghai), College of Food Science and Technology, Shanghai Ocean University, Shanghai 201306, China; 2 Shanghai Agricultural Product Quality and Safety Center (Shanghai), Shanghai 200125, China

**Keywords:** microfluidic paper-based analytical devices, detection, chlorpyrifos, profenofos, cypermethrin

## Abstract

Chlorpyrifos (CHL), profenofos (PRO) and cypermethrin (CYP) are widely used in combination to increase crop yields. However, these three pesticides can cause serious harm to human health and do not easily degrade. In this study, a novel visible paper sensor has been prepared successfully and different colorimetric reactions were utilized to detect the three pesticides simultaneously. The sensor was constructed by grafting a zwitterionic polymer onto a cellulose filter (CF) and placing it on a glass surface modified with PDMS. The branch shape was designed to form multiple detection areas, which were modified with specific pesticides and corresponding chromogenic reagents. The as-prepared colorimetric platform exhibited high sensitivity, a short detection time, a good linear response and a low detection limit (LOD) for the three pesticides (chlorpyrifos: y = 46.801 − 1.939x, R^2^ = 0.983, LOD = 0.235 mg/L; profenofos: y = 40.068 + 42.5x, R^2^ = 0.988, LOD = 4.891 mg/L; cypermethrin: y = 51.993 + 1.474x, R^2^ = 0.993, LOD = 4.053 mg/L). The comparison of the results obtained by the proposed paper sensor and those obtained by spectrophotometry further revealed the stability and reliability of the paper sensor. In particular, the color intensity of the interaction between the pesticides and coloring agents could be directly observed by the human eye. The consistency of the colorimetric/optical assay was proven in real target pesticide samples. Thus, this sensing strategy provides a portable, cost-effective, accurate and visualized paper platform, which could be suitable for application in the fruit and vegetable industry for monitoring CHL, PRO and CYP in parallel.

## 1. Introduction

Pesticides are commonly used in the production of vegetables, fruits and grains, because most of them are highly effective and less resistant. However, it cannot be ignored that the widespread use of pesticides could lead to pesticide residues over the maximum residue limit (MRL) according to relevant standards. Excessive pesticide residues in the ecosystem will cause serious pollution to water and soil, thus affecting human safety [1,2,3]. Therefore, it is indispensable to develop a simple and effective method for detecting pesticide residues in agricultural products [4]. In recent years, there were some efficient analytical methods developed to detect pesticide concentrations in food and water, including gas chromatography (GC) [5,6], high-performance liquid chromatography (HPLC) [7] and gas chromatography–tandem mass spectrometry (GC/MS) [8]. Although these analytical technologies display higher sensitivity and accuracy, they still have drawbacks, such as complex operations and expensive equipment, which greatly limit the real-time detection of pesticide residues.

Since the first microfluidic paper-based analytical devices (μPAD) reported by Whitesides (2008) for biosensing, paper devices have exhibited the anticipated performance in point-of-care testing fields [9,10,11]. This method provides tremendous advantages in terms of cost-effectiveness, high throughput and fast response times [12,13]. Meanwhile, it can be integrated with colorimetry to establish a visual device, which facilitates the development of portable devices for in-field food analysis [14,15]. With the development of compound pesticides, the variety of pesticides on a single sample is increasing [16]. For example, chlorpyrifos (CHL), profenofos (PRO) and cypermethrin (CYP), which are frequently used in combination to improve crop resistance in weeds and insect pests for increasing crop yields, should be tested simultaneously [17]. These three pesticides are highly toxic and do not easily degrade. The residues of them on food will cause pollution to the environment and pose threats to the human immune and nervous systems [18,19,20,21]. Due to this concern, maximum residue limits for these three pesticides in various crops have been established in many countries [22]. According to the China National Standard System (GB 2763-2021), the maximum residual limits of chlorpyrifos, profenofos and cypermethrin in fruits and vegetables are 3 mg/kg (<0.235 mg/L), 10 mg/kg (<4.891 mg/L) and 7 mg/kg (<4.053 mg/L), respectively, which are all larger than the detection limits of the sensor. The use of μPAD makes it possible to detect different types of compound pesticide residues, which greatly improves the detection efficiency and reduces the cost of analytes. However, bare paper sensors are susceptible to interference caused by non-specific proteins, microbes and biofilms, leading to limited application in complex real samples. Moreover, the sensitivity and quantification ability of bare paper sensors cannot satisfy the requirements of trace detection [23,24]. Therefore, it is of great importance to address this issue for the better realization of on-site testing with high sensitivity.

In fact, the modification of a cellulose filter (CF) with hydrophilic polymers, such as poly (L-lactic acid) [25] and poly (ethylene glycol) [26,27], has been commonly used to improve the permeability of water and suppress the adhesion of contaminants. Recently, zwitterionic polymers, including poly(sulfobetaine methacrylate) (pSBMA), have been proven to be promising anti-fouling hydrophilic materials owing to their excellent performance, with good biocompatibility and high stability [28,29]. Liu et al. [30] developed a functionalized thin film using zwitterionic polymers and silver nanoparticles (AgNPs). The obtained membranes showed excellent performance in bacterial inactivation. Sun et al. [31] reported a preparation strategy for novel hybrid ultrafiltration membranes via a polysulfone casting solution of UiO-66-pSBMA in a phase inversion method. The ultrafiltration membranes with UiO-66-pSBMA presented a fast exchange rate between solvent and nonsolvent. Li et al. [32] summarized the recent studies on the improved hydrophilicity, antifouling and antibacterial properties of various inorganic/organic surfaces grafted with zwitterionic polymers. According to this review, we found that the cellulose filter (CF) with zwitterionic polymers was rarely studied in the sensor detection field.

In this research, we established an economical pSBMA-functionalized colorimetric multichannel paper-based analytical device for the identification and quantitation of three pesticides in complex samples. By using atom transfer radical polymerization (ATRP), pSBMA was grafted onto the surface of a cellulose filter (CF). The pSBMA-CF possesses the advantages of ultra-low fouling and superior hydrophilicity.

The functionalized pSBMA-μPAD could detect analytes in a complicated matrix without a large number of reagents. When the reaction occurred on the device, the error of detection condition and operation were minimized by matching the absorbance and color relative intensity with the three pesticides’ concentrations. Finally, the pSBMA-μPAD was successfully applied to determine the three target pesticides in real vegetable and fruit samples and the reliability was confirmed by spectrum analysis. The proposed pSBMA-μPAD platform may provide a cost-effective, simple and accurate method for the rapid quantitative detection of pesticide content in food.

## 2. Materials and Methods

### 2.1. Materials and Instruments

Whatman filter paper No. 1 (150-mm diameter) was purchased from Whatman International Ltd. (Shanghai, China). Acetylcholinesterase (AChE, 222 units/mg from electric ell), ninhydrin, 4-aminoantipyrine, acetylthiocholine iodide (ATChI), 5,5-dithiobis(2-nitrobenzoic) acid (DTNB), chlorpyrifos, profenofos, cypermethrin, β-propiolactone (95%), copper(II) bromide (CuBr_2_, 98%), cuprous(I) bromide (CuBr, 98%) 2-bromisobutyryl bromide (BIBB, 98%), 2,2′-bipyridine (BPY), bromoisobutyryl bromide (C_4_H_6_Br_2_O), 11-hydroxy-1-undecanethiol (C_11_H_24_OS), triethylamine (TEA, 99%), tetrahydrofuran (THF, HPLC grade) and 3-[dimethyl-[2-(2-methylprop-2-enoyloxy) ethyl] azaniumyl] propane-1-sulfonate (SBMA) were obtained from Sigma-Aldrich (Shanghai, China). Dichloromethane (CH_2_Cl_2_), methanol (CH_3_OH), sodium hydroxide (NaOH), ethanol (CH_3_CH_2_OH), acetone (CH_3_COCH_3_), isopropanol (CH_3_CHOHCH_3_), hydrochloric acid (HCl) and ethyl ether (CH_3_CH_2_OCH_2_CH_3_) were provided by Sinopharm Chemical Reagent Co., Ltd. (Shanghai, China). Polydimethylsiloxane (PDMS; Sylgard 184) was purchased from Dow Corning. Ammonium acetate (CH_3_COONH_4_) and potassium ferricyanide (K_3_[Fe(CN)_6_]) were obtained from Aladdin Industrial Inc. (Shanghai, China). Phosphate-buffered saline (PBS) and bovine serum albumin (BSA) were provided by Solarbio (Beijing, China). All solutions used were compounded by ultrapure water.

The FT-IR measurements were performed on an FT-IR spectrometer (Thermo Scientific, Waltham, MA, USA). All XPS spectra were identified by X-ray photoelectron spectroscopy (XPS, Thermo Scientific EscaLab 250Xi, USA). The UV–vis spectrum and absorbance were measured using a UV–vis absorption spectrophotometer (Shimadzu Corporation, UV-vis-2550, Kyoto, Japan).

### 2.2. Preparation of Standard and Reagent Solution

The stock solutions of 1000 mg/L chlorpyrifos, profenofos and cypermethrin were prepared with methanol, respectively. These solutions were diluted with ultrapure water to obtain the diluents with a series of concentration gradients. ATChI (0.2 g) was dissolved in ultrapure water (100 mL) to prepare ATChI solution (0.2% *w*/*v*). AChE (800 units) was dissolved in PBS (100 mL) to prepare AChE solution (8 units/mL). DTNB (0.08 g) was dissolved in PBS (100 mL) to prepare DTNB solution (0.08% *w*/*v*). Potassium ferricyanide (2.0 g) was dissolved in ultrapure water (100 mL) to prepare potassium ferricyanide solution (2% *w*/*v*). 4-aminoantipyrine (1.5 g) was dissolved in ultrapure water (100 mL) to prepare 4-aminoantipyrine solution (1.5% *w*/*v*). Ninhydrin (3 g) was dissolved in ethyl alcohol (100 mL) to prepare ninhydrin solution (3% *w*/*v*). Ammonium acetate (2.5 g) was dissolved in ultrapure water (100 mL) to prepare ammonium acetate solution (2.5% *w*/*v*).

### 2.3. Fabrication of the pSBMA-μPAD

**Figure 1:**
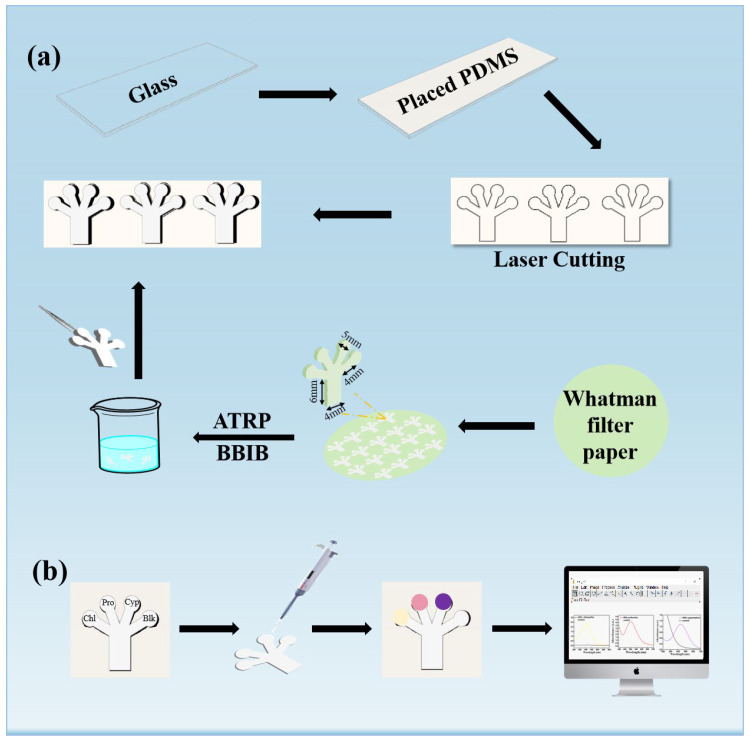
(**a**) The design for fabrication of pSBMA-μPAD; (**b**) process of the μPAD-based simultaneous analysis of chlorpyrifos, profenofos and cypermethrin detection.

The hydrophilic and hydrophobic interface was manufactured to prevent the interference of non-target substances and improve the stability of the reaction system. The μPAD was prepared as follows (Figure 1a). ① The preparation of the hydrophobic channel: First, the glass surface was modified with allyltrimethoxysilane, and then solidified with PDMS to compose hydrophobic barriers, and finally operated with a laser cutter to create the designed paper shape on the glass. ② The fabrication of pSBMA-coated cellulose filter (pSBMA-CF): The hydrophilic channel was designed by AutoCAD, and it was mainly composed of a dendritic reaction channel (5-mm diameter) and a straight channel (the width was 4 mm and the length was 6mm). By using an ATRP reaction, the pSBMA was grafted onto the surface of the designed paper. The details of the grafting reaction are presented in Figure S1. ③ After the pSBMA-CF was placed on the patterned PDMS channel, the pSBMA-μPAD assay platform was successfully established. To verify whether pSBMA was successfully grafted onto the surface of CF, the changes in the surface’s elemental composition before and after grafting with pSBMA were observed by XPS and FT-IR.

### 2.4. Colorimetric Analysis

Figure 1b shows the method by which the μPAD was used for the simultaneous detection of the three pesticides. Firstly, the pSBMA-μPAD was pretreated with the corresponding buffer solution and chromogenic reagent and dried for 3 min at room temperature. Then, the pesticide detection was performed by adding 2.0 μL of standard solution or real sample to the test zone. After the reaction, ImageJ software was used to obtain the gray intensity of the image. Image processing and data acquisition are explored and discussed in the Supplementary Materials. A linear curve between the mean relative intensity and the corresponding analyte concentration was established, which was employed for the quantitative analysis of the pesticides in real samples.

### 2.5. Real Sample Testing

To evaluate the performance of the established sensor in practical applications, five vegetables were used as test samples to determine the residual amounts of the three pesticides (CHL, PRO and CPY). Meanwhile, nine vegetables were selected to verify the practicability of the method. According to the China National Standard System (GB 23200.93-2016 and GB/T 5009.110-2003), the sample pretreatment was performed. Briefly, each sample (20.0 g) was homogenized and then extracted with acetonitrile (40 mL). After centrifugation, the supernatant (4 mL) was further filtered with a microporous porosity filter (0.22 μm) to avoid the interference of impurities. Each sample was added with two concentrations (10 mg/L and 20 mg/L) of the three pesticides (CHL, PRO and CPY), and tested with the pSBMA-μPAD at least three times.

## 3. Results

### 3.1. XPS Measurements

**Figure 2:**
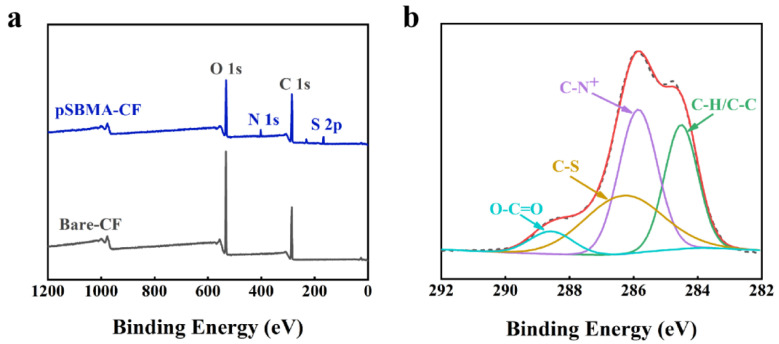
(**a**) The XPS spectra of bare-CF and pSBMA-CF; (**b**) representative XPS high-resolution C1s spectra of pSBMA-CF.

Figure 2a presents the difference in XPS spectra between the bare-CF and pSBMA-CF. It was found that the bare-CF included the peaks of C 1s (286.4 eV) and O 1s (532.8 eV), which agreed well with the elemental composition of CF. There was no signal of N in the bare-CF, while a new peak at 401.9 eV (corresponding to N 1s) was seen in the XPS spectrum of pSBMA-CF. Another new, weak peak at 167.2 eV was assigned to S 2p (Figure S2), which was attributed to the oxidation of S, such as sulfonate (-SO_3_^−^) [33]. After graft copolymerization, the XPS C 1s spectrum of pSBMA-CF (Figure 2b) was curved-fitted into four peaks at 284.5, 285.7, 286.3, and 288.9 eV, owing to C-C/C-H, C-N^+^, C-S, and O-C=O, respectively [34,35,36,37]. According to the XPS data in Table S1, after functionalization, the signal of carbon was increased, while the signal of oxygen decreased, and small amounts of nitrogen (3.95%, N 1s) and sulfur (3.62%, S 2p) were detected. These results indicated that the pSBMA polymer was successfully grafted onto the CF by the ATRP reaction.

### 3.2. FT-IR Analysis

**Figure 3:**
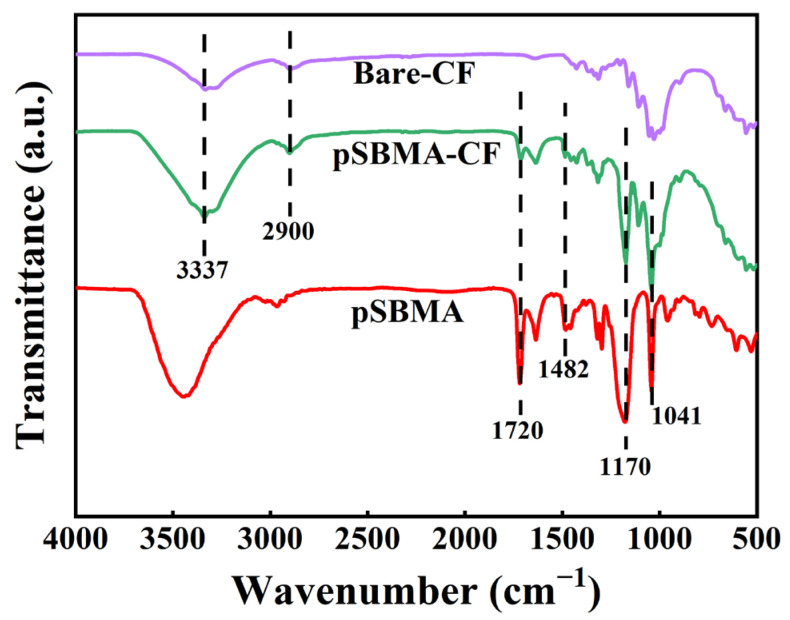
The FT-IR spectra of pSBMA, bare-CF and pSBMA-CF.

With respect to the FT-IR spectrum of bare-CF and pSBMA-CF, the bare-CF spectrum showed the characteristic peaks highlighted in Figure 3 in purple (wide stretching vibration of -OH at 3337 cm^−1^, and skeletal vibration of C-H at 2900 cm^−1^) [38]. Moreover, the FT-IR spectrum of pSBMA-CF (Figure 3 green) not only possessed characteristic peaks with CF, but also showed absorption peaks consistent with pSBMA (Figure 3 red). Compared with the bare-CF, there were two new peaks at 1482 cm^−1^ and 1720 cm^−1^ in the FTIR spectra of the pSBMA-CF, which were attributed to the quaternary ammonium and carbonyl groups in SBMA, respectively. The other two absorption peaks at 1041 cm^−1^ and 1170 cm^−1^ were assigned to the S=O symmetric stretch and the S=O asymmetric stretch of the sulfonate groups of SBMA, respectively [39]. In short, the FI-TR results confirmed that pSBMA was successfully grafted with bare-CF via ATRP.

### 3.3. Evaluating Hydrophobicity, Hydrophilicity and Sensing Properties

**Figure 4:**
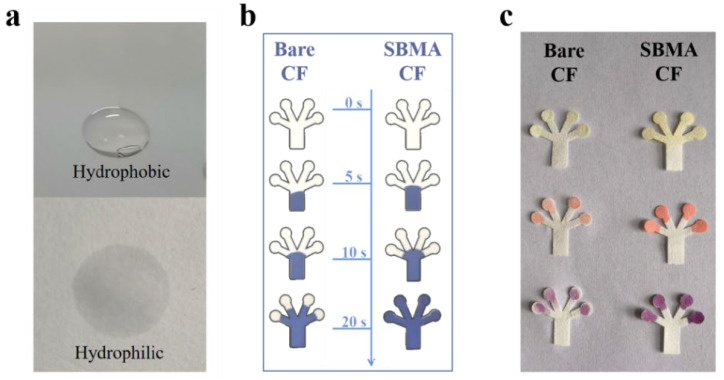
(**a**) Water dropped onto hydrophobic and hydrophilic zones; (**b**) the time dependence of the adsorption of blue ink on bare-CF and pSBMA-CF, respectively; (**c**) the detection of chlorpyrifos, profenofos and cypermethrin using bare-CF and pSBMA-modified CF.

The same amount of water (2 μL) was dropped onto PDMS-modified glass (hydrophobic area) and pSBMA-modified filter paper (hydrophilic area), respectively, to evaluate the hydrophilic and hydrophobic properties of the platform. Figure 4a shows that the water drop placed in the hydrophobic zone formed its expected arc-shaped surfaces with the glass, while the water placed on the filter paper immediately infiltrated the paper as expected. Moreover, the difference in hydrophilicity between pSBMA-CF and bare-CF was compared, by dropping 2 µL of blue ink onto the paper chip to monitor the ink’s movement over time (Figure 4b). Compared with the unmodified CF, the pSBMA-CF exhibited better directional wettability, and the blue ink filled the whole pSBMA-CF in only 20 s. Finally, chlorpyrifos, profenofos and cypermethrin were used to verify whether the pSBMA-modified CF is beneficial in terms of its sensing ability (Figure 4c). For an independent comparison of each nutrient, images were captured after the same number of standard solutions were added to the reaction zone for 3 min. Compared with the bare-CF, the pSBMA-CF displayed prominent color intensity for each pesticide, indicating that the pSBMA-CF can effectively improve the sensing ability.

### 3.4. Reaction Principle of the Three Pesticides

**Figure 5:**
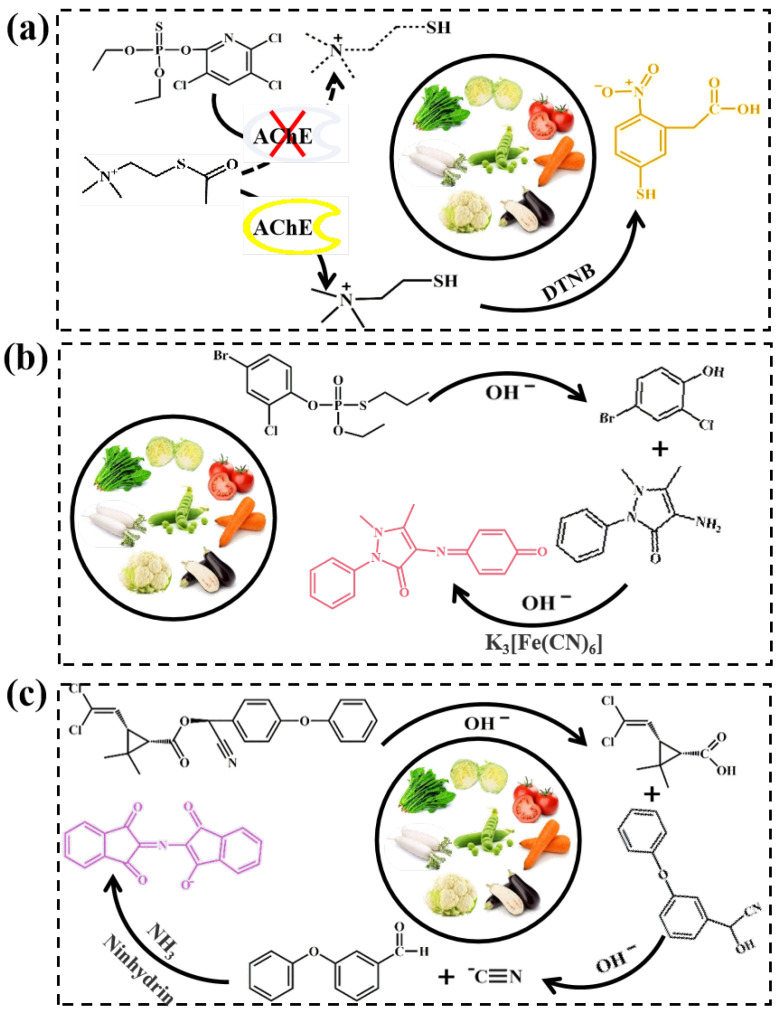
The reaction principle of (**a**) chlorpyrifos, (**b**) profenofos and (**c**) cypermethrin.

The principle of chlorpyrifos analysis is depicted in Figure 5a. In the presence of acetylcholine molecules, AChE catalyzed the formation of choline, which reacted with DTNB, resulting in the formation of TNB with a yellow color. The unique inhibitory effect of chlorpyrifos on AChE activity decreased the TCh production, and the yellow intensity was gradually reduced to colorless [40]. The higher the concentration of chlorpyrifos, the stronger the inhibition, and then the weaker the intensity of the yellow signal perceived by the sensor. A schematic of the detection of profenofos is displayed in Figure 5b. Profenofos was first hydrolyzed by NaOH to generate 2-chloro-4-bromophenol, which further reacted with potassium ferricyanide and 4-aminoantipyrine to produce a unique red chelate. Ninhydrin, a well-known color reagent for detecting cyanide, is commonly used to identify and quantify cyanide compounds [41]. A schematic of the cypermethrin experiment is depicted in Figure 5c. Under alkaline conditions, cypermethrin was hydrolyzed to generate cyanide, which further decomposed to free cyanide ions. The cyanide ions then reacted with ninhydrin and ammonium acetate to form a purple compound.

### 3.5. Colorimetric Detection for Chlorpyrifos

**Figure 6:**
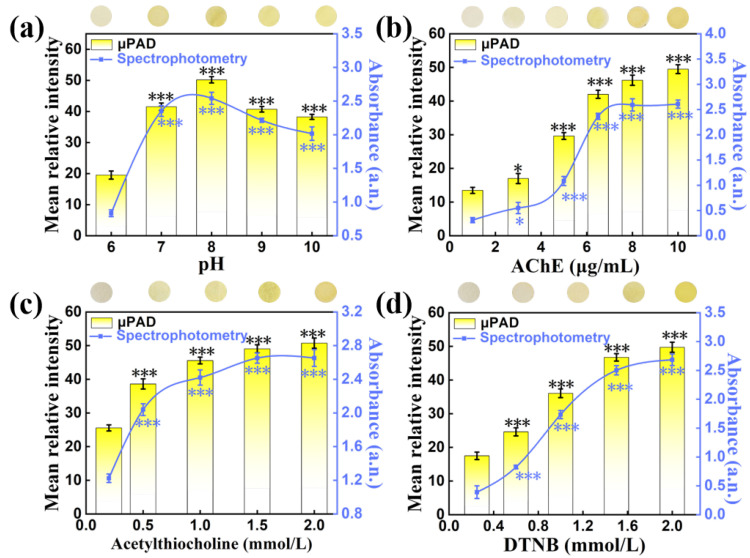
The effects of experimental conditions on pSBMA-μPAD and spectrophotometry: (**a**) pH of system buffer, (**b**) the concentration of ATChI, (**c**) the concentration of AChE and (**d**) the concentration of DTNB. “*” stands for significance test.

**Figure 7:**
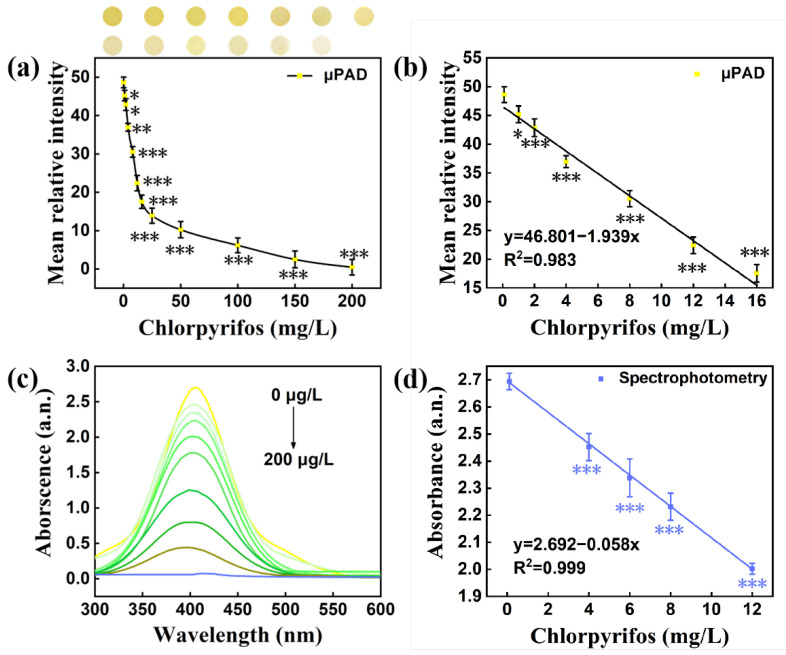
(**a**) The effect of different chlorpyrifos concentrations on the mean relative intensity of pSBMA-μPAD; (**b**) the correlation between the chlorpyrifos concentration and the mean relative intensity of pSBMA-μPAD; (**c**) absorbance of different chlorpyrifos concentrations at different wavelengths; (**d**) the chlorpyrifos concentration correlation between concentration and absorbance. “*” stands for significance test.

The buffer pH, time and the concentration of DTNB, ATChI and AChE on pSBMA-μPAD were optimized to achieve the best color reaction. In order to find the solution environment with the highest acetylcholinesterase activity, the pH value was adjusted from 6 to 10. As can been observed in Figure 6a, the mean relative intensity of μPAD was proven to be the optimal at pH = 8. Moreover, we found that the color of this system started to stabilize after 10 min (Figure S3). As a result, the optimal conditions (pH 8, 10 min) were chosen in subsequent experiments. AChE catalyzes the hydrolysis of ATChI, which was the key to transform DTNB into TNB. As shown in Figure 6b, the mean relative intensity increased with the increasing concentration of AChE, and tended to be stable after 6 μg/mL. According to the previous work, the chromogenic reagent had a great effect on color development. The chromogenic reagent was prepared by mixing different concentrations of ATChI (0–2 mmol/L) and DTNB (0–2 mmol/L) in equal volumes (1:1 ratio). The mean relative intensity of both ATChI and DTNB was positively correlated with the concentration, and tended to be stable when the concentration reached 2 mmol/L (Figure 6c,d). Therefore, ATChI (2 mM) and DTNB (2 mM) were selected to pretreat the reaction region to ensure the accuracy of the subsequent experiment. According to the above optimal conditions (pH 8.0, AChE of 6 μg/mL, ATCh of 2 mM, DTNB of 2mM), the correlation equation y = 46.801 − 1.939x (y, mean relative intensity; x, chlorpyrifos concentration, mg/L) between the chlorpyrifos concentration and color intensity in the range of 0.1–16 mg/L was obtained, and the correlation coefficient R^2^ value reached 0.983 (Figure 7a,b). The limit of detection (LOD, S/N = 3) for chlorpyrifos was 0.235 mg/L. Concurrently, the comparison experiments were accomplished with spectrophotometry, and all data were acquired and analyzed at the characteristic peak (408nm). Under the optimal experimental conditions, a good linear equation (y = 2.692 − 0.058x, R^2^ = 0.999, LOD = 0.091 mg/L) was obtained (Figure 7c,d). Compared with spectrophotometry, the pSBMA-μPAD greatly improves the convenience and the detection is faster.

### 3.6. Colorimetric Detection for Profenofos

**Figure 8:**
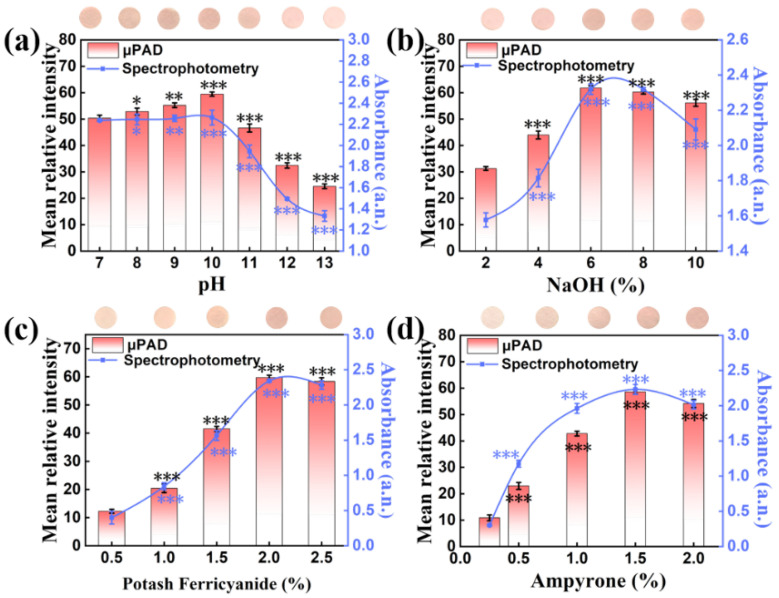
The effects of experimental conditions on pSBMA-μPAD and spectrophotometry: (**a**) the concentration of NaOH, (**b**) pH of system buffer, (**c**) the concentration of potassium ferricyanide and (**d**) the concentration of 4-aminoantipyrine. “*” stands for significance test.

**Figure 9:**
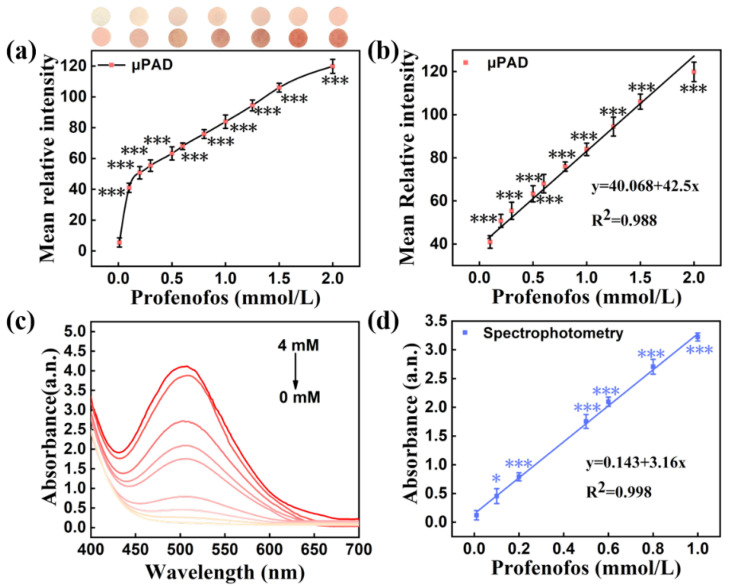
(**a**) The effect of different profenofos concentrations on the mean relative intensity of pSBMA-μPAD; (**b**) the correlation between the profenofos concentration and the mean relative intensity of pSBMA-μPAD; (**c**) absorbance of different profenofos concentrations at different wavelengths; (**d**) the profenofos concentration correlation between concentration and absorbance. “*” stands for significance test.

To successfully perform this reaction on pSBMA-μPAD, the optimization of the pH, NaOH, potassium ferricyanide and 4-aminoantipyrine was carried out, respectively. As manifested in Figure 8a, the mean relative intensity was almost unchanged when the pH was 7.0–10.0, but the complexation reaction was obviously inhibited when the pH was over 10.0. Therefore, pH 10.0 was selected as the optimal pH for subsequent experiments. In addition, the mean relative intensity increased and achieved the maximum when the NaOH concentration (Figure 8b) reached 6%, the potassium ferricyanide concentration (Figure 8c) reached 2% and the 4-aminoantipyrine concentration (Figure 8d) reached 1.5%. Moreover, the unique color signal achieved a stable stage after 10 min of reaction (Figure S4). Hence, all the above optimized conditions were selected to pretreat the detection area. On the pretreated area, a linear relationship over the range of 0.08–2 mM was obtained between the chlorpyrifos concentration and color intensity, with a correlation equation of y = 40.068 + 42.5x and a correlation coefficient R^2^ = 0.988 (Figure 9a,b). A detection limit as low as 4.891 mg/L was achieved. Additionally, the analysis of profenofos was confirmed by spectrophotometry, and all data were captured at a 508 nm characteristic peak. Under the optimal experimental conditions (pH 10.0, NaOH 6%, potassium ferricyanide 2%, 4-aminoantipyrine 1.5%), a good linear equation for profenofos (y = 0.143 + 3.16x, R^2^ = 0.998, LOD = 0.118 mg/L) was obtained (Figure 9c,d). All the above results indicate that the pSBMA-μPAD has great potential value in rapid testing.

### 3.7. Colorimetric Detection for Cypermethrin

**Figure 10:**
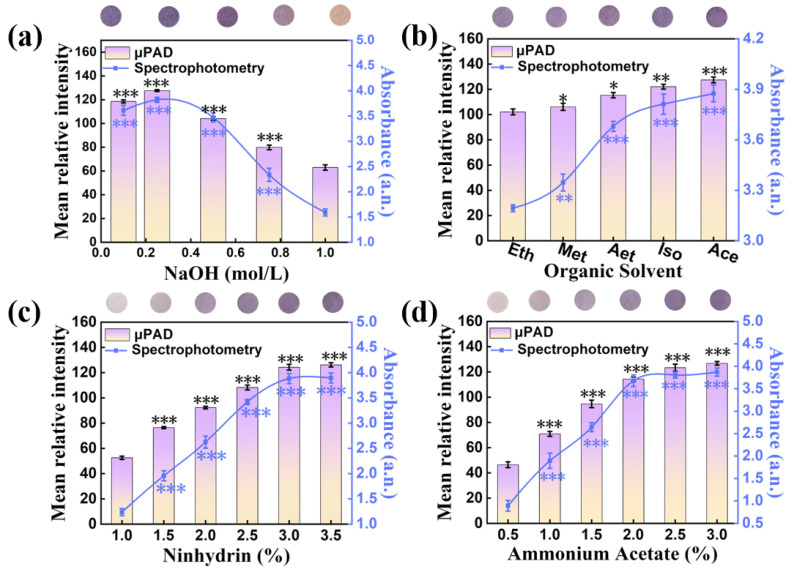
The factors affecting the experimental results on pSBMA-μPAD and spectrophotometry: (**a**) the concentration of hydrolytic solution, (**b**) the hydrolytic solution system, (**c**) the concentration of ninhydrin and (**d**) the concentration of ammonium acetate. “*” stands for significance test.

**Figure 11:**
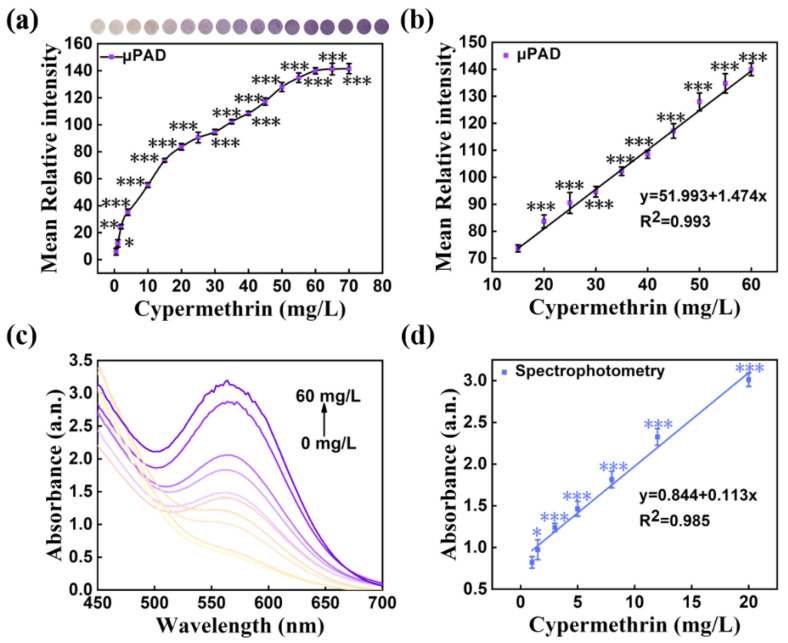
(**a**) The effect of different cypermethrin concentrations on the mean relative intensity of pSBMA-μPAD; (**b**) the correlation between the cypermethrin concentration and the mean relative intensity of pSBMA-μPAD; (**c**) absorbance of different cypermethrin concentrations at different wavelengths; (**d**) the cypermethrin concentration correlation between concentration and absorbance. “*” stands for significance test.

The hydrolysis system was composed of NaOH solution and organic solvents, and the organic solvents could increase the extent of hydrolyzation. To ensure the full reaction of this experiment on pSBMA-μPAD, the concentration of NaOH and the type of organic solution were optimized. When the NaOH concentration was 0.25 mol/L, the purple signal was the highest (Figure 10a). Moreover, as shown in Figure 10b, compared with ethanol (Eth), methanol (Met), aether (Aet), isopropanol (Iso) and acetone (Ace) could accelerate the hydrolyzation better and resulted in a stronger purple signal. Subsequently, it was found that the color of this system started to stabilize after 20 min of reaction (Figure S5). These experimental conditions (NaOH 0.25 mol/L, acetone, 20 min) were chosen for subsequent color reagent optimization. As seen in Figure 10c,d, with the increasing amounts of ninhydrin and ammonium acetate, the color intensity increased notably and tended to be stable when ninhydrin reached 3% and ammonium acetate was over 2%. Consequently, 3% ninhydrin and 2% ammonium acetate were chosen to prepare the color reagent for further tests. According to the above optimal conditions, a good linear response was acquired at the range of 12.0 to 60.0 mg/L, with a correlation equation of y = 51.993 + 1.474x, and the correlation coefficient R^2^ value reached 0.993 (Figure 11a,b). The detection limit of cypermethrin obtained was as low as 4.053 mg/L. Correspondingly, the absorbance data of cypermethrin were collected from the characteristic peak at 570 nm, and a good linear equation (y = 0.844 + 0.113x, R^2^ = 0.985, LOD = 1.17 mg/L) was established (Figure 11c,d). According to the above results, the pSBMA-μPAD not only has excellent detection performance for cypermethrin, but also displays high consistency with the spectrophotometry results.

### 3.8. Application of the μPAD to Detection in Real Samples

To assess the actual detection performance of pSBMA-μPAD in fruit and vegetable samples, five fresh fruits and vegetables (apple, orange, spinach, tomato, cucumber) were selected as samples. The rapid detection of chlorpyrifos, profenofos and cypermethrin in fruits and vegetables was carried out in the form of adding the target. At the same time, a UV–visible spectrophotometer was used to realize the additional verification of the detection results. As shown in Table 1, Table 2 and Table 3, the relative recoveries of pSBMA-μPAD for CHL, PRO and CYP were 93.5–105.7%, 94.5–108.3% and 90.9–108.4%, respectively. The results were highly consistent with the results obtained by UV–visible spectrophotometry, indicating that pSBMA-μPAD could successfully detect pesticides in fruits and vegetables. Meanwhile, nine vegetables were selected to verify the practicability of the method. The CHL solution, PRO solution and CYP solution were configured at 0, 0.5, 1 and 2 times the detection limit concentration to treat the actual samples. Among them, 0 and 0.5 times were negative samples, and 1 and 2 times were positive samples, which were added to the detection area for the color reaction. Table 4 shows that the system could clearly distinguish negative and positive samples, and it had high accuracy and practicability.

## 4. Discussion and Conclusions

In summary, we have proposed a method for the efficient detection of three commonly used pesticides in fruits and vegetables using a portable and affordable multiplexed pSBMA-μPAD. On the paper-based detection platform, each pesticide was detected by reacting with its own selective substrates. The concentrations of three pesticides were derived from the color intensity using ImageJ software. After the experimental conditions were optimized, the limits of detection (LOD) of CHL, PRO and CYP were 0.235, 4.891 and 4.053 mg/L using the paper-based colorimetric microfluidic device, respectively. The pSBMA-μPAD constructed through the combination of colorimetric methods and a paper sensor is an important step toward the determination and quantification of trace pesticide residues, which was demonstrated using various samples of vegetables. Thus, this cheap and portable paper chip provides a promising method for the on-site assay of pesticide residues with high sensitivity and selectivity.

## Figures and Tables

**Table 1:** The detection of CHL in five fruit and vegetable samples by pSBMA-μPAD and UV–visible spectrophotometry.

Samples	Added (mg/L)	CHL Concentration (mg/L)		RSD (%) (n = 3)	
		μPAD	Spectrophotometry	μPAD	Spectrophotometry
Apple	10	9.56	9.88	4.9	3.7
	20	20.23	20.01	2.8	3.1
Orange	10	10.57	9.94	3.8	4.3
	20	19.73	21.19	3.6	3.1
Spinach	10	10.07	9.77	2.9	3.2
	20	20.09	19.97	4.2	3.1
Tomato	10	9.88	9.52	5.6	4.9
	20	20.19	19.03	2.3	4.7
Cucumber	10	9.35	9.81	5.1	3.6
	20	20.56	19.32	2.9	3.4

**Table 2:** The detection of PRO in five fruit and vegetable samples by pSBMA-μPAD and UV–visible spectrophotometry.

Samples	Added (mg/L)	PRO Concentration (mg/L)		RSD (%) (n = 3)	
		μPAD	Spectrophotometry	μPAD	Spectrophotometry
Apple	10	10.83	10.07	5	4.7
	20	20.21	20.63	3.9	4.3
Orange	10	9.59	10.55	2.6	4.1
	20	19.86	20.06	3.3	3.8
Spinach	10	9.45	9.77	5.1	4.5
	20	20.39	19.56	6.7	4.2
Tomato	10	10.38	9.41	3.5	2.9
	20	19.92	21.08	3.7	6.2
Cucumber	10	9.79	10.28	5.2	4.8
	20	20.02	21.32	7.3	4.2

**Table 3:** The detection of CYP in five fruit and vegetable samples by pSBMA-μPAD and UV–visible spectrophotometry.

Samples	Added (mg/L)	CYP Concentration (mg/L)		RSD (%) (n = 3)	
		μPAD	Spectrophotometry	μPAD	Spectrophotometry
Apple	10	9.79	10.88	5.7	4.6
	20	19.75	20.36	2.9	3.4
Orange	10	9.99	10.58	2.7	3.3
	20	20.32	20.76	4.5	6.1
Spinach	10	10.84	10.23	5.5	2.1
	20	21.07	20.51	3.5	3.8
Tomato	10	9.09	10.36	7.2	4.8
	20	20.95	19.08	1.9	2.8
Cucumber	10	9.79	10.32	3.7	4.9
	20	18.91	20.04	6.2	6.8

**Table 4:** Verification of the accuracy of pSBMA-μPAD in the detection of CHL, PRO and CYP in 15 fruit and vegetable samples.

	Pesticides	CHL (LOD)				PRO (LOD)				CYP (LOD)			
Samples		0	0.5	1	2	0	0.5	1	2	0	0.5	1	2
Baby cabbage		−	−	+	+	−	−	+	+	−	−	+	+
White radish		−	−	+	+	−	−	+	+	−	−	+	+
Lettuce		−	−	+	+	−	−	+	+	−	−	+	+
Carrot		−	−	+	+	−	−	+	+	−	−	+	+
Spinach		−	−	+	+	−	−	+	+	−	−	+	+
Celery		−	−	+	+	−	−	+	+	−	−	+	+
Lettuce		−	−	+	+	−	−	+	+	−	−	+	+
Pterocladia tenuis		−	−	+	+	−	−	+	+	−	−	+	+
Bok choy		−	−	+	+	−	−	+	+	−	−	+	+
Cabbage		−	−	+	+	−	−	+	+	−	−	+	+
Broccoli		−	−	+	+	−	−	+	+	−	−	+	+
Cucumber		−	−	+	+	−	−	+	+	−	−	+	+
Apple		−	−	+	+	−	−	+	+	−	−	+	+
Orange		−	−	+	+	−	−	+	+	−	−	+	+
Tomato		−	−	+	+	−	−	+	+	−	−	+	+

“−” means negative and “+” means positive.

## Data Availability

The data presented in this study are available on request from the corresponding author.

## Supplementary Materials

The following supporting information can be downloaded at: https://www.mdpi.com/article/10.3390/bios13030309/s1, Figure S1: Reaction scheme for grafting pSBMA onto CF [42]; Figure S2: Representative XPS high-resolution S2p spectra of pSBMA-CF [43,44]; Figure S3: The effect of time for the detection of chlorpyrifos by pSBMA-μPAD; Figure S4: The effect of time for the detection of profenofos by pSBMA-μPAD; Figure S5: The effect of time for the detection of cypermethrin by pSBMA-μPAD; Table S1: Relative compositions of bare-CF and pSBMA-CF-modified cellulose filter measured by XPS (Atomic Concentration, %).
