# Supplementary material for: Simultaneous and Sensitive Detection of Three Pesticides Using a Functional Poly(Sulfobetaine Methacrylate)-Coated Paper-Based Colorimetric Sensor
Source: Biosensors (Basel). 2023 Feb 22;13(3):309. doi: 10.3390/bios13030309 (PMC10046087; doi:10.3390/bios13030309)
Supplement: Supplementary file 1 [file biosensors-13-00309-s001.zip › biosensors-2124339-supplementary.pdf]

biosensors

MDPI

Supplementary Material

Simultaneous and Sensitive Detection of Three Pesticides Using a Functional Poly(Sulfobetaine Methacrylate)-Coated Paper-Based Colorimetric Sensor

Matrials and methods

Grafting pSBMA onto cellulose paper surface.

In order to obtain a highly sensitive assay platform, 3-[dimethyl-[2-(2-methylprop-2-enoyloxy) ethyl] azaniumyl] propane-1-sulfonate (sulfobetaine methacrylate, SBMA) was grafted onto the surface of the paper device via atom transfer radical polymerization (ATRP), referring to a previous work [1]. Firstly, we synthe-sized the ARTP initiator (ω-mercaptoundecyl bromoisobutyrate): bromoisobutyryl bromide (0.41 mL, 3.34 mmol) was added drop by drop to a stirred solution of 11-mercapto-1-undecanol (0.75 g, 3.67 mmol) and pyridine (0.27 mL, 3.34 mmol) dissolved in anhydrous dichloromethane. The reaction was stirred at 0 °C for 1 h and then at room temperature for 16 h. After this, the mixture was added to water and the product was extracted with toluene. Then, the extract was washed with ether and saturated ammonium chloride, and finally dried by sodium sulfate. Secondly, for the immobilization of the initiator, the cellulose filter (CF) was washed with acetone and tetrahydrofuran (THF), and thereafter sonicated in both solvents. As shown in Fig. S1, the ATRP initiator for the ATRP reaction with SBMA was then immobilized on the surface of the CF through esterification of the hydroxyl group with 2-bromoisobutyryl bromide (BIBB). The CF substrate was first im-mersed in THF (20 mL) solution containing TEA (148 mg, 1.46 mmol) and catalytic DMAP, and then BIBB (305 mg, 1.33 mM) was added for the reaction. The reaction was implemented overnight in a shaking appa-ratus at room temperature. After completion of the reaction, the filter paper substrate was subsequently rinsed with EtOH and THF to sequentially remove unbound polymer and by-products. The filter paper was lastly kept and dried in a vacuum oven overnight at 45°C. Thirdly, the polymers were grafted onto the CF surface using ATRP. Before the polymerization, the CF was washed with ethanol and THF, respectively, and then the dried CF was immersed in 1 mM ATRP initiator in an ethanol solution at room temperature for 24 h. For typical ATRP polymerization, CuBr, $CuBr_2$, BPY, deoxygenated methanol and the CF coated with initiators were placed in a reaction tube under nitrogen protection. Then, the SBMA monomer was dissolved in deoxy-genated methanol (6 mL) and water (2 mL), and we transferred the solution to the reaction tube with a deox-ygenated needle tube under the protection of nitrogen. The ATRP reaction proceeded for a predetermined period. After polymerization, the pSBMA-functionalized CF was removed from the solution; washed thor-oughly with ethanol, PBS and distilled water ultrasonically; and dried under a vacuum before use.

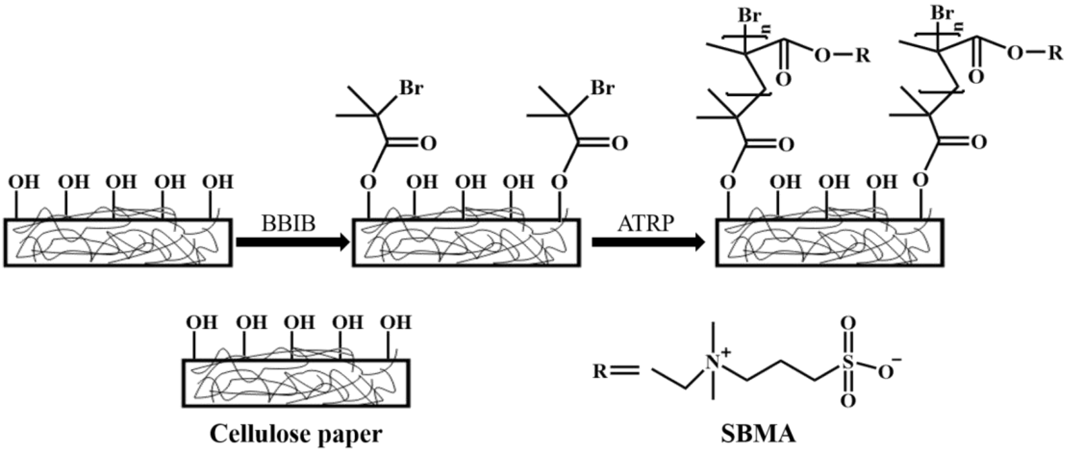

Figure S1. Reaction scheme for grafting pSBMA onto CF.

Characterization.

For the spectrum of the pSBMA-grafted paper, a new peak S 2p (≈167.2 eV) was detected during the measurement, which was attributed to the sulfobetaine moiety of the grafted pSBMA [2]. The S 2p1 (≈168 eV) and S 2p3 (≈166.9 eV) peaks were exhibited in the high-resolution spectrum of S 2p (Fig. S2), which was attributed to the sulfonate groups in pSBMA [3].

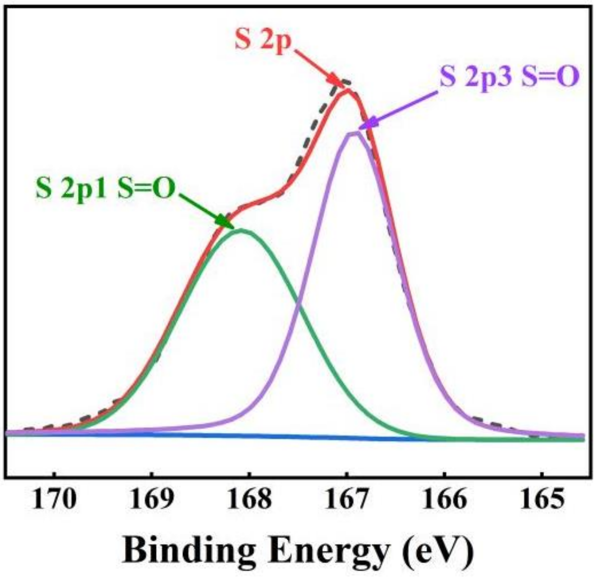

Figure S2. Representative XPS high-resolution S2p spectra of pSBMA-CF.

Table S1. Relative compositions of bare-CF and pSBMA-CF-modified cellulose filter measured by XPS (Atomic Concentration, %).

	Bare-CF	pSBMA-CF
C 1s	60.04	62.26
O 1s	39.96	30.17
N 1s	—	3.95
S 2p	—	3.62

Note: "—" indicates no detection

Detection of chlorpyrifos, profenofos and cypermethrin with pSBMA-μPAD.

Chlorpyrifos assay.

Firstly, the standard and vegetable samples were incubated with AChE enzyme for 3 min at 37 °C. Then, we dropped the incubation solution onto the chlorpyrifos sensing area of pSBMA-μPAD, which was pretreated with PBS buffer solution (pH 8.0), ATChI solution (2 mM, 1μL) and DTNB solution (2 mM, 1μL). The chlorpyrifos standard solutions ranging from 0.1 mg/L to 16 mg/L were allowed to build linear curves for further analysis.

Profenofos assay.

Under the optimum conditions, the pSBMA-μPAD was pretreated with borate buffer solution (pH = 10) and a chromogenic reagent. The chromogenic reagent was created by mixing 1.5 % (w/v) 4-amino antipyrine and 2% (w/v) potassium ferricyanide together with a volume ratio of 1:1. Then, the profenofos standard solutions at different concentrations (0.08 mmol/L~2 mmol/L) were used to build linear curves for further analysis.

Cypermethrin assay.

Under the optimum conditions, the chromogenic agent was composed of ninhydrin (3.5%) and ammonium acetate (3%) in a volume ratio of 1:1. After adding the hydrolytic solution standard solutions or vegetable samples, a good linear equation of cypermethrin was built over the range of 12.0–60.0 mmol/L for further analysis.

Image processing and data acquisition.

All photos were captured by a digital camera and processed with ImageJ. The camera was fixed on a simple tripod and operated at a distance of 20 cm from the paper device in automatic mode. Moreover, the images were captured before and after exposure to the pesticide. For each sensing area, the changes in color intensity were calculated by ImageJ software. The software converted the color intensity of the captured images into three numerical mean values corresponding to the color elements of red, green and blue. The difference between the color values of the before and after photos was calculated by the following equation:

$$\Delta R = \bar{R}_{after} - \bar{R}_{before}$$

$$\Delta G = \bar{G}_{after} - \bar{G}_{before}$$

$$\Delta B = \bar{B}_{after} - \bar{B}_{before}$$

In this equation, the difference values of red, green and blue color elements are denoted by ΔR, ΔG and ΔB, respectively. Next, the difference values between the reaction point and the blank point were re-converted to gray intensity by the following equation:

$$\Delta Gray = 0.30\Delta R + 0.59\Delta G + 0.11\Delta B$$

Based on the above formula, the mean relative intensity was obtained from the RGB readings measured in the reference area and sensing area after each experiment was repeated three times.

Statistical data analyses were performed in the Microsoft Excel software package (Microsoft, Redmond, WA, USA) and Origin 2019b (OriginLab, Northampton, MA, USA).

Detection for chlorpyrifos.

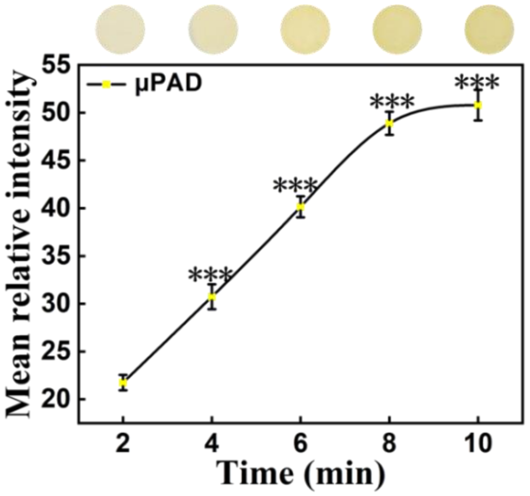

Figure S3. The effect of time for the detection of chlorpyrifos by pSBMA-μPAD.

Detection for profenofos.

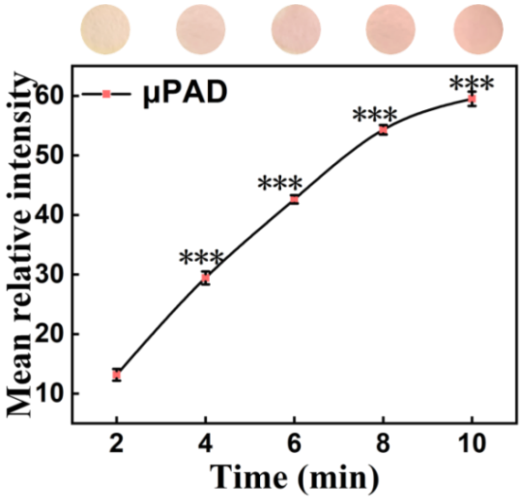

Figure S4. The effect of time for the detection of profenofos by pSBMA-μPAD.

Detection for cypermethrin.

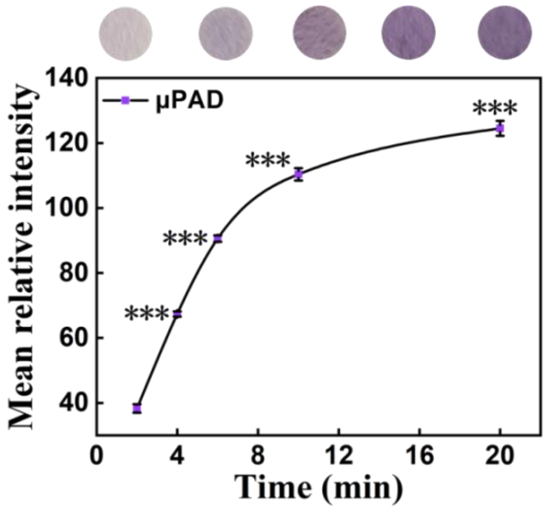

Figure S5. The effect of time for the detection of cypermethrin by pSBMA-μPAD.

References

1. Cheng, Y., Wang, J.L.; Li, M.L.; Fu, F.F.; Zhao, Y.; Yu, J. Zwitterionic polymer-grafted superhydrophilic and superoleophobic silk fabrics for anti-oil applications. Macromol. *Rapid Commun*. **2020,** *41,* 2000162.
2. Jones, D.M., Brown, A.A.; Huck, W.T.S. Surface-initiated polymerizations in aqueous media: effect of initiator density. *Langmuir* **2002,** *18,* 1265-1269.
3. Lamberti, A.; Serrapede, M.; Ferraro, G.; Fontana, M.; Perrucci, F.; Bianco, S.; Chiolerio, A.; Bocchini, S. All-SPEEK flexible supercapacitor exploiting laser-induced graphenization. *2D Mater*. **2017,** *4,* 035012.
